# A preliminary biomechanical study on trachea reconstruction surgery using the clavicular periosteum

**DOI:** 10.3389/fbioe.2023.1117483

**Published:** 2023-01-17

**Authors:** Xiaoli Zhu, Kangli Sun, Xin Xia, Yu Chen, Anqiang Sun, Xingming Chen

**Affiliations:** ^1^ Department of Otolaryngology-Head and Neck Surgery, Peking Union Medical College Hospital, Peking Union Medical College and Chinese Academy of Medical Sciences, Beijing, China; ^2^Key Laboratory for Biomechanics and Mechanobiology of Ministry of Education, Beijing Advanced Innovation Center for Biomedical Engineering, School of Biological Science and Medical Engineering, Beihang University, Beijing, China; ^3^ Department of Radiology, Peking Union Medical College Hospital, Peking Union Medical College and Chinese Academy of Medical Sciences, Beijing, China

**Keywords:** trachea, reconstruction surgery, finite element, airway mechanics, clavicular periosteum

## Abstract

**Introduction:** The clavicular periosteum is a suitable material for trachea reconstruction. However, because the periosteum is softer and has different mechanical properties than tracheal cartilage, the mechanical loads under physiological conditions after trachea reconstruction may cause collapse or stenosis of the repaired trachea.

**Methods:** In this study, the mechanical properties of the clavicular periosteum were tested, and the 3D trachea geometry was constructed based on CT-scanning images acquired before the surgery. Differing degrees of stenosis (0%, 33%, and 55%) for the repaired trachea sections were predetermined, presenting the different degrees of the tracheal cross-sectional area immediately after clavicular periosteum reconstruction. Then the biomechanical environments of the trachea and the airflow were simulated and analyzed.

**Results:** In the fluid mechanics simulation, the air pressure on the patch area decreased with increasing degrees of stenosis, while the fluid velocity increased as stenosis increased. In solid mechanics simulations, patch area deformation increased as the cross-sectional area of the trachea decreased, and the stress in the patch increased as stenosis increased.

**Discussion:** The solid stress changes may cause tissue remodeling, thickening, and scarring of the patch area. The fluid mechanical changes in the repaired trachea would further aggravate the stenosis. The numerical simulation study would provide references for biomechanical evaluation of trachea reconstruction surgery. The surgical indications may be expanded in the future based on the model prediction results.

## 1 Background

Invasion of the trachea affects approximately 6% of patients with primary thyroid cancer and 10% of recurrent cases (Brauckhoff 2014). Complete trachea wall resection provides better oncological outcomes than tangential incomplete wall resection in most invasive cases (Honings et al., 2010; Matsumoto and Ikeda 2021). A large portion of trachea wall invasions is non-circular. Window resection can achieve radical resection in most non-circular invasion cases (Brauckhoff 2014). The sternocleidomastoid muscle (SCM) myoperiosteal flap is a suitable option for one-stage reconstruction of tracheal or laryngotracheal non-circular defects (Xia et al., 2021). This flap contains the SCM muscle that can be used as the pedicle and the clavicular periosteum as the reconstructive tissue. The clavicular periosteum has a natural curvature similar to the tracheal wall curve with bone regeneration capacity. However, the periosteum is softer than the tracheal cartilage, which may cause tracheal wall deformation and changes in air fluid dynamics in the tracheal lumen. Patients experiencing this complication may suffer from dyspnea due to cross-sectional area loss. Most of the literature suggests that the SCM myoperiosteal flap is safe for defects that measure no more than half of the tracheal circumference (Ebihara et al., 2011; Matsumoto and Ikeda 2021). However, current methods of tracheal reconstruction are highly dependent on the surgeon’s experience, causing much uncertainty about the post-operative patency of the trachea. Hence, an objective approach to preoperatively predict the tracheal cross-sectional area loss is essential.

Biomechanical modeling is a non-invasive physiological simulation technology that can objectively analyze mechanical features and tracheal deformation by computational simulation analysis. Biomechanical models have been used to simulate the deformation of the cardiovascular wall and blood flow in the cardiovascular lumens (Sommer et al., 2018; Gao et al., 2020). Computational fluid dynamics (CFD) has been used in some tracheal stenosis and tracheomalacia patients (Gunatilaka et al., 2020; Poynot et al., 2020). However, simulation analysis of tracheal wall deformation after reconstruction has yet to be reported.

In this study, we attempted to establish a preliminary objective evaluation method to estimate the risk of postoperative tracheal stenosis after trachea reconstruction with clavicular periosteum (SCM as the pedicle). A biomechanical model of tracheal repair using the clavicular periosteum was established in two patients. The fluid environment of the repaired trachea and the mechanical deformation of the trachea wall were analyzed in multiple circumstances with various levels of cross-sectional area retention, representing different outcomes after surgery.

## 2 Materials and methods

### 2.1 Clavicular periosteum tensile analysis

**FIGURE 1:**
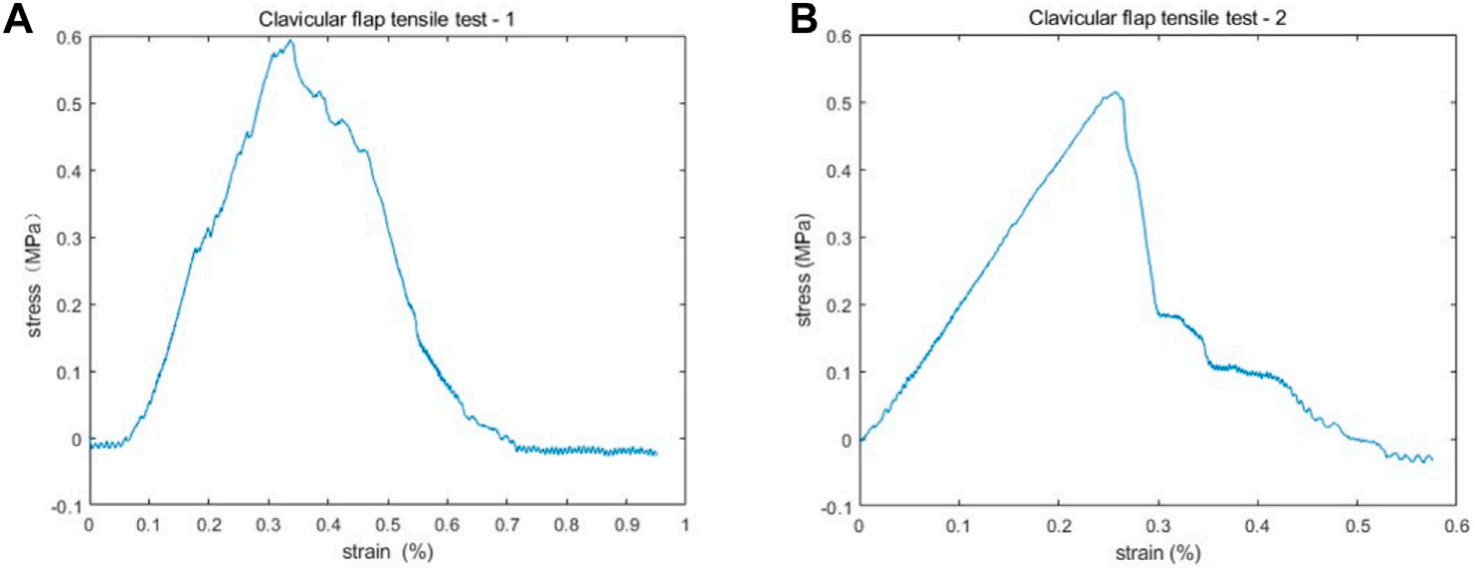
The stress-strain results of two clavicular periosteum samples after uniaxial tensile testing. **(A,B)** The stress-strain curves are highly consistent. In the initial phase of stretching, the stain increases with stress linearly. The elastic modulus of the two periostea was 2.68 MPa **(A)** and 2.53 MPa **(B)**, respectively.

A 2 cm by 2 cm clavicle periosteum section was obtained intraoperatively from a male patient with recurrent laryngeal cancer invading the sternoclavicular joint. The specimen was immediately placed in saline and transported at room temperature to the laboratory for uniaxial tensile testing, as previously reported in the literature (Safshekan et al., 2019 and Trabelsia et al., 2009). The muscle tissue was removed from the sample, the periosteum was divided into two equal parts, and tensile tests were performed along the long axis of each sample. Using a Rigel micro-controlled electronic universal testing machine, the samples were subjected to uniaxial tensile tests at room temperature. Each sample was first subjected to a pretreatment step to obtain reproducible results. Then uniaxial tensile tests were performed at a strain rate of 5 mm/min until failure while being moisturized by a saline solution. The force-displacement curves were highly consistent, as shown in Figure 1. During the initial phase of stretching, the clavicular periosteum produced a linear elastic deformation. By fitting the linear region of the experimental data, the elastic modulus of the two clavicular periostea was calculated as 2.68 and 2.53 MPa, respectively. The average of the two, 2.6 MPa, was used as the elastic modulus of the clavicular periosteum. Due to the limitation of experimental conditions, Poisson’s ratio of the clavicular periosteum was set to 0.33 with reference to Tsai et al. (2010).

### 2.2 Clinical parameters

#### 2.2.1 Patient general information

In order to illustrate the different mechanical conditions of the reconstructed trachea, we selected two typical patients with significant differences in the cross-sectional area of the trachea and postoperative effects for simulation.

Patient one was a 51-year-old man with a previous right thyroid papillary carcinoma treated with bilateral thyroidectomy, neck dissection, and adjuvant radioactive Iodine 131 treatment. The patient presented 2 years and 8 months after initial treatment with a mass in the right thyroid area that invaded three tracheal rings and bilateral cervical lymph node metastasis. He underwent bilateral neck dissection, remnant thyroidectomy, tracheal window resection (partial cricoid cartilage and four tracheal rings) and SCAM flap reconstruction.

Patient two was a 44-year-old woman with a previous right thyroid papillary carcinoma that was treated with a right thyroidectomy. The patient presented 7 years after initial treatment with a right thyroid papillary carcinoma invading two tracheal rings. She underwent a total thyroidectomy, tracheal window resection (four tracheal rings), and SCAM flap reconstruction.

#### 2.2.2 Fibrolaryngoscopic evaluation

A fibrolaryngoscopic evaluation was performed on each patient before the surgery, immediately after the surgery in the operation room, and every month for 1 year after surgery. After administering the dicaine topical anesthetic, the patients were asked to lay supine on the examination bed. The video laryngoscope was inserted through the nose, and the trachea was observed in a state of calm breathing.

#### 2.2.3 Imaging

A 3T computed tomography (CT) scan (Siemens Somatom Definition Flash, Siemens Healthcare, Erlangen, Germany) of the neck and chest was performed on each patient before the operation. During the scanning process, the patients were asked to lay flat in the CT scanning chamber, keeping the head and body stable. The scanning layer thickness was 0.4 mm, and the scanning time was 33.25 s.

#### 2.2.4 Surgery

**FIGURE 2:**
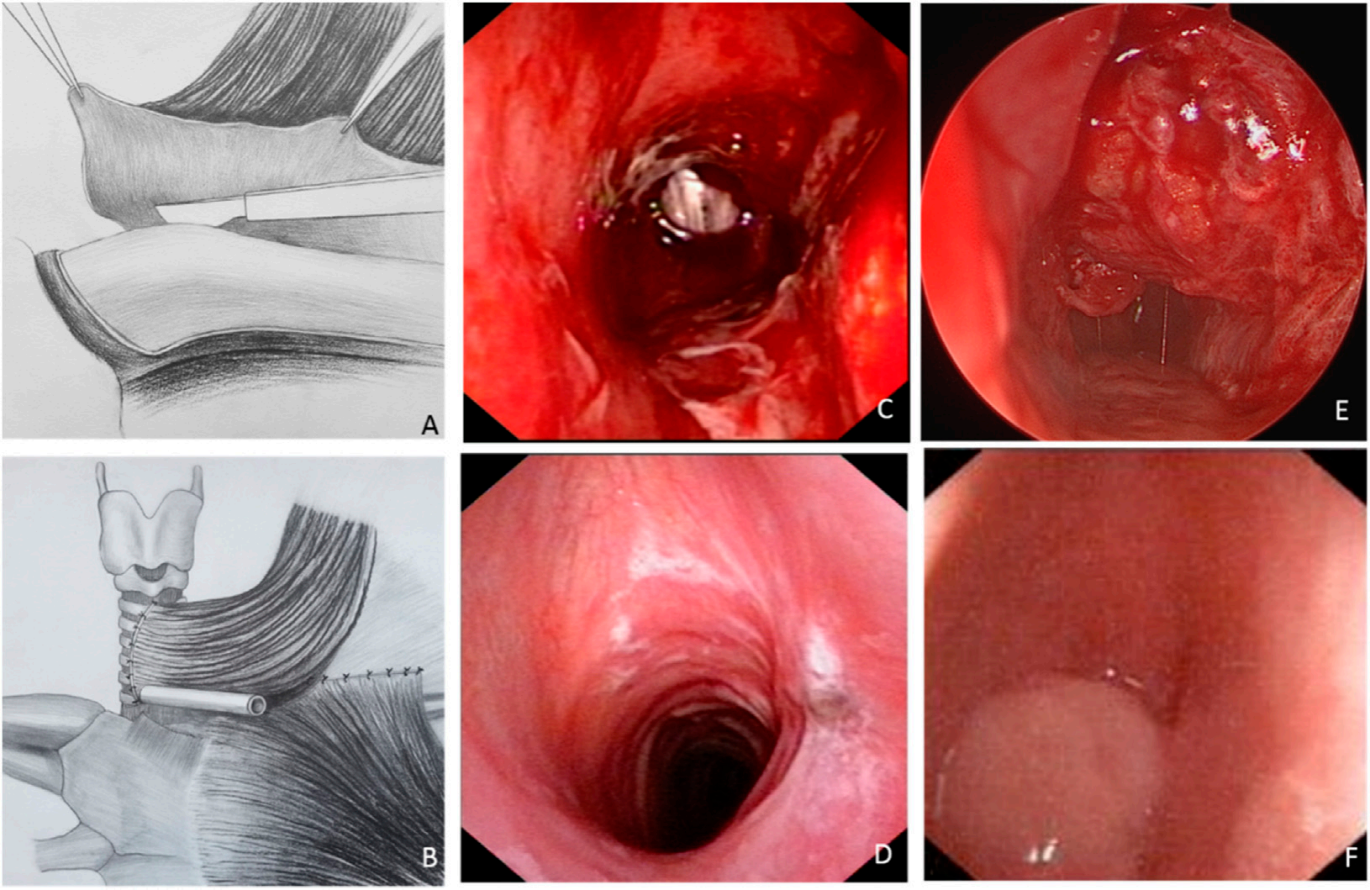
The tracheal reconstruction process *via* the sternocleidomastoid muscle (SCM) myoperiosteal flap and the laryngoscopic images of the two patients. **(A,B)** The clavicular periosteum was harvested and transposed to repair the tracheal defect. **(C,D)** Laryngoscopic images of patient one taken immediately **(C)** and 2-month **(D)** after surgery. **(E,F)** Laryngoscopic images of patient two taken immediately **(E)** and 2-month **(F)** after surgery.

For each patient, a window resection of the trachea (with or without part of the larynx) was performed, and a clavicular periosteal flap was harvested for reconstruction, with the SCM used as the pedicle (Figures 2A, B). The defect included partial cricoid cartilage and four tracheal rings in the first patient and four tracheal rings in the second patient. The widths for both patients were half of the cross-sectional area of the trachea. The fibrolaryngoscopic images of each patient immediately after surgery are shown in Figures 2C, E. The tracheal cross-sectional area of the first patient appeared close to normal (Figure 2C), while about half of the normal tracheal area was observed in the second patient (Figure 2E).

#### 2.2.5 Trachea reconstruction model

**FIGURE 3:**
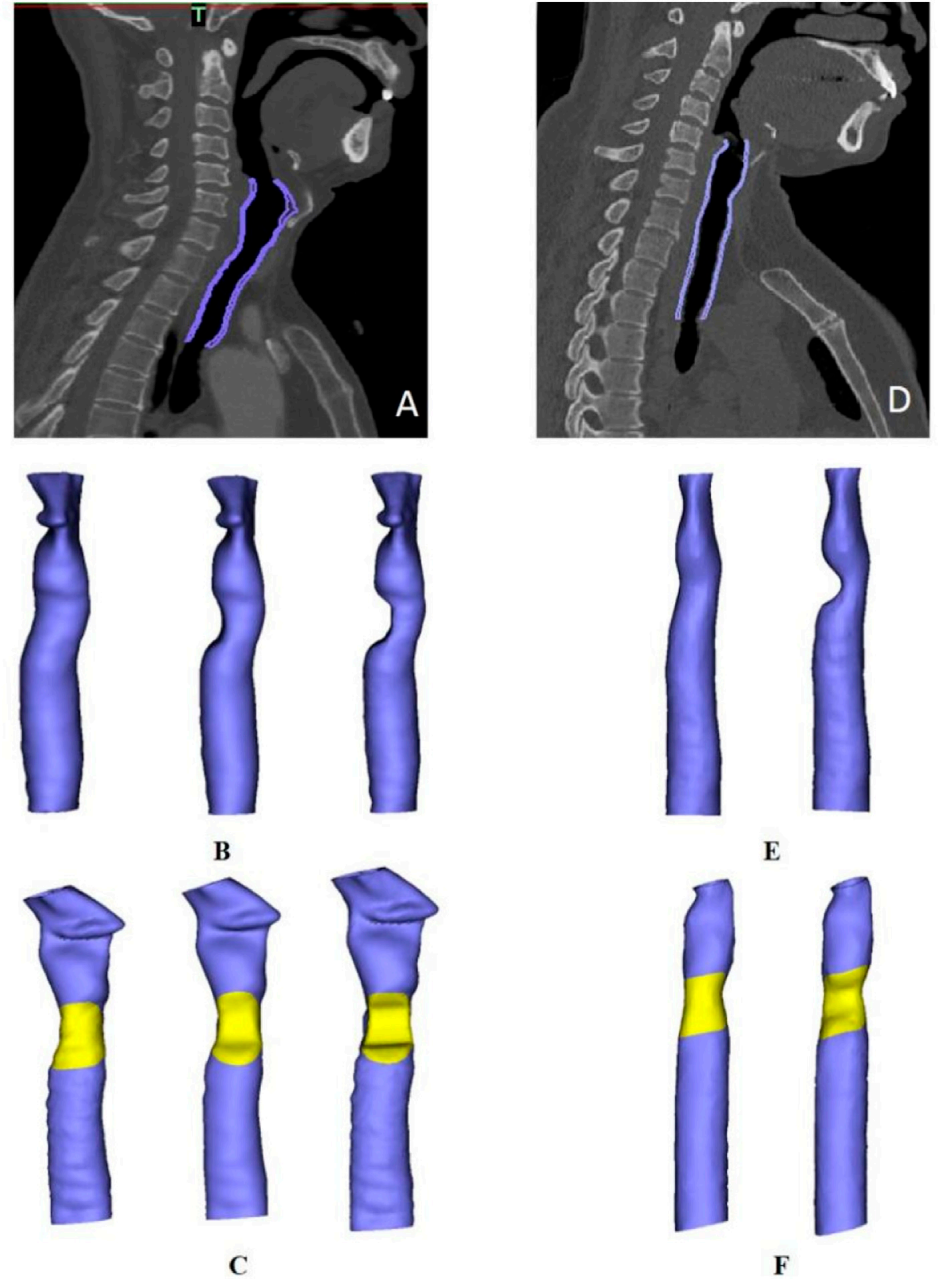
Trachea model reconstruction in two patients. **(A,D)** Purple areas represent the scope of the model in patients one and two, respectively. **(B)**. Three tracheal models for patient one with different degrees of stenosis (from left to right: normal, 33% stenosis and 50% stenosis). **(C,F)** Diagram of patch positioning in each patient. **(E)**. Models depicting a normal trachea and a trachea with 50% stenosis in patient two.

Digital Imaging and Communications in Medicine (DICOM) data from preoperative tracheal CT scans were imported into Mimics software for 3D reconstruction of the tracheal wall. A tracheal model from the subglottis to the innominate artery was established. The extent of the clavicle periosteum patch was determined according to the actual size of the intraoperative tracheal defect (Figure 3). In addition, to simulate different degrees of post-operative trachea lumen stenosis, trachea models with various degrees of stenosis were established according to the laryngoscope images. The position of the patch was marked in yellow.

#### 2.2.6 Tidal volume and respiratory rate measurement

The pulmonary function tests were performed using portable spirometry (Homebreath, Shenzhen, China) in a quiet status preoperatively. The tidal volume and respiratory rate of the patients’ calm breathing were recorded four times, the results were averaged (Table 1), and the testing time was 5 min.

**TABLE 1:** The tidal volume (VT) of the two patients.

	VT1(L)	VT2(L)	VT3(L)	VT4(L)	Average VT(L)
Patient 1	0.76	0.54	0.53	0.57	0.60
Patient 2	0.54	0.53	0.56	0.52	0.54

L: litre.

### 2.3 Fluid mechanics simulation

#### 2.3.1 Assumptions and governing equations

For this study, the air inside the trachea was assumed to be incompressible, homogeneous and Newtonian fluid. The fluid domain was governed by the Navier-Stokes equation (Eq. 1) and the continuity equation (Eq. 2):

$$\rho \frac{\partial \vec{u}}{\partial t}+\rho \left(\vec{u}•\nabla \right)\vec{u}+\nabla p-\mu \nabla^{2}\vec{u}=0$$ (1)

$$\nabla \cdot \vec{u}=0$$ (2)

where p and $\vec{u}$ represent pressure and fluid velocity vector, respectively. The density of air (ρ) was 1.225 kg·m^−3^ with a constant viscosity (μ) of 1.84 × 10^–5^ Pa·s. The steady K-epsilon standard model also was used in this study.

The Fluent module in Ansys 19.2 (ANSYS, Inc., Canonsburg, PA, United States) was used for the simulation. Airflow velocity was calculated using the SIMPLE algorithm, and the pressure-based solver was selected for the pressure correction and the sequential solution of the momentum equation. The discrete windward format was used with second-order accuracy. The maximum root mean square residual was set to 10^–5^.

#### 2.3.2 Mesh generation

Tetrahedral meshing elements were chosen due to the irregular shape of the fluid model. The uniform model of the normal trachea consisted of 1,747,572 elements, the trachea with 33% stenosis consisted of 1,636,223 elements, and the trachea with 50% stenosis consisted of 1,550,360. The dimensions of the mesh were 0.3 mm.

#### 2.3.3 Boundary conditions

Based on the measured respiratory rate and tidal volume, the inlet velocity was calculated as 3.1 m/s in the first patient and 4 m/s in the second patient. The outlet was defined as Outflow. The walls were concluded to be rigid and no-slip.

### 2.4 Solid mechanics simulation

#### 2.4.1 Material properties

In this study, the isotropic linear elastic model was used to define the physical properties of the clavicle periosteum, and the elastic modulus of the periosteum was set to 2.6 MPa based on the results of the tensile test. Additionally, Poisson’s ratio was set to 0.33 with reference to the nasal bone periosteum (Tsai et al., 2010). The isotropic elastic model was also used to define the tracheal wall. The material property parameters of the tracheal cartilage were defined as the material properties of the tracheal wall. The elastic modulus of the patient’s tracheal cartilage ring was calculated with a magnitude of 16.5 MPa (Safshekan et al., 2016), and Poisson’s ratio was set to 0.49 (Trabelsi et al., 2009).

#### 2.4.2 Mesh generation

Tetrahedral elements were used for mesh division of the periosteum patch and the trachea, with element sizes of 0.3 and 0.5 mm, respectively.

#### 2.4.3 Load and solution

The intrapulmonary pressure of the human body during calm inspiration was used as the negative pressure load to be applied to the tracheal wall in this study. A uniform pressure of 2 mmHg was applied to the lateral wall of the trachea (Wilson and Theodore, 2016). Both ends of the trachea model were fixed. The contact between the patch and the trachea was bonded.

## 3 Results

### 3.1 Clinical results

As previously mentioned, the tracheal cross-sectional area of the first patient was close to normal immediately after surgery (Figure 2C), and there was no obviously compromised area after healing (Figure 2D). While the initial tracheal cross-sectional area of the second patient was about half of the normal tracheal area (Figure 2E), severe tracheal stenosis and granuloma were observed 2 months after surgery (Figure 2F).

### 3.2 Fluid simulation results

**FIGURE 4:**
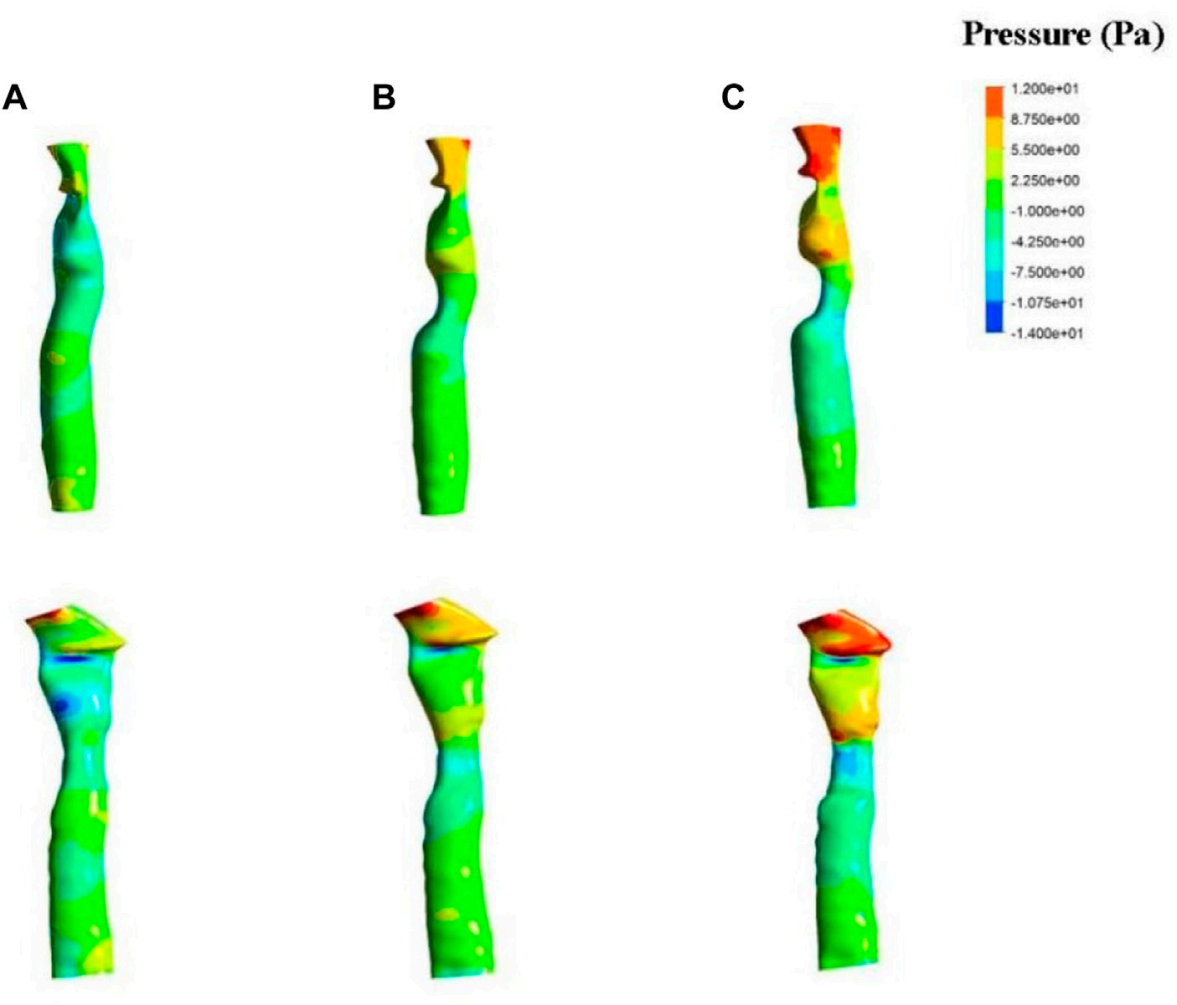
Results of fluid pressure in patient one. The anterior view (top) and lateral view (bottom) of a normal trachea **(A)**, a trachea with 33% stenosis **(B)** and a trachea with 50% stenosis **(C)**.

The fluid simulation analysis for patient one (Figure 4) revealed the effect of different degrees of stenosis on the pressure distribution in and around the patch area. The pressure distribution under normal morphology was uniform, and the pressure on the medial side of the tracheal wall was between −4 and 2 Pa, which is consistent with the study of Ruiz (Ruiz and Aristizabal, 2017). However, the pressure on the trachea with 33% stenosis ranged from −1–7 Pa, and the minimum value was found near the wall, where the pressure was −7.5 Pa. A lower pressure was observed on the trachea with 50% stenosis, ranging from −1 to −10 Pa, and the lowest pressure was −10.7 Pa near the wall.

The fluid simulation analysis of the second patient (Figures 6A–C) exhibited an internal pressure of between 0 and 10 Pa in the trachea with normal morphology. With 50% stenosis, the pressure ranged from −20 to 10 Pa, and the negative pressure was even lower with a minimum value of −40 Pa. Compared with the results from the first patient, the negative pressure in the second patient was greater at the same degree of stenosis.

In addition, changes in pressure above the patch area for the first patient were observed with different degrees of stenosis. Increased stenosis resulted in positive pressure in the area above the patch, and the positive pressure value also increased. A positive pressure of 2–5 Pa was observed in the 33% stenosis trachea, which increased to 8.75 Pa at 50% stenosis. The pressure above the stenosis for the second patient increased as well compared to the normal trachea.

**FIGURE 5:**
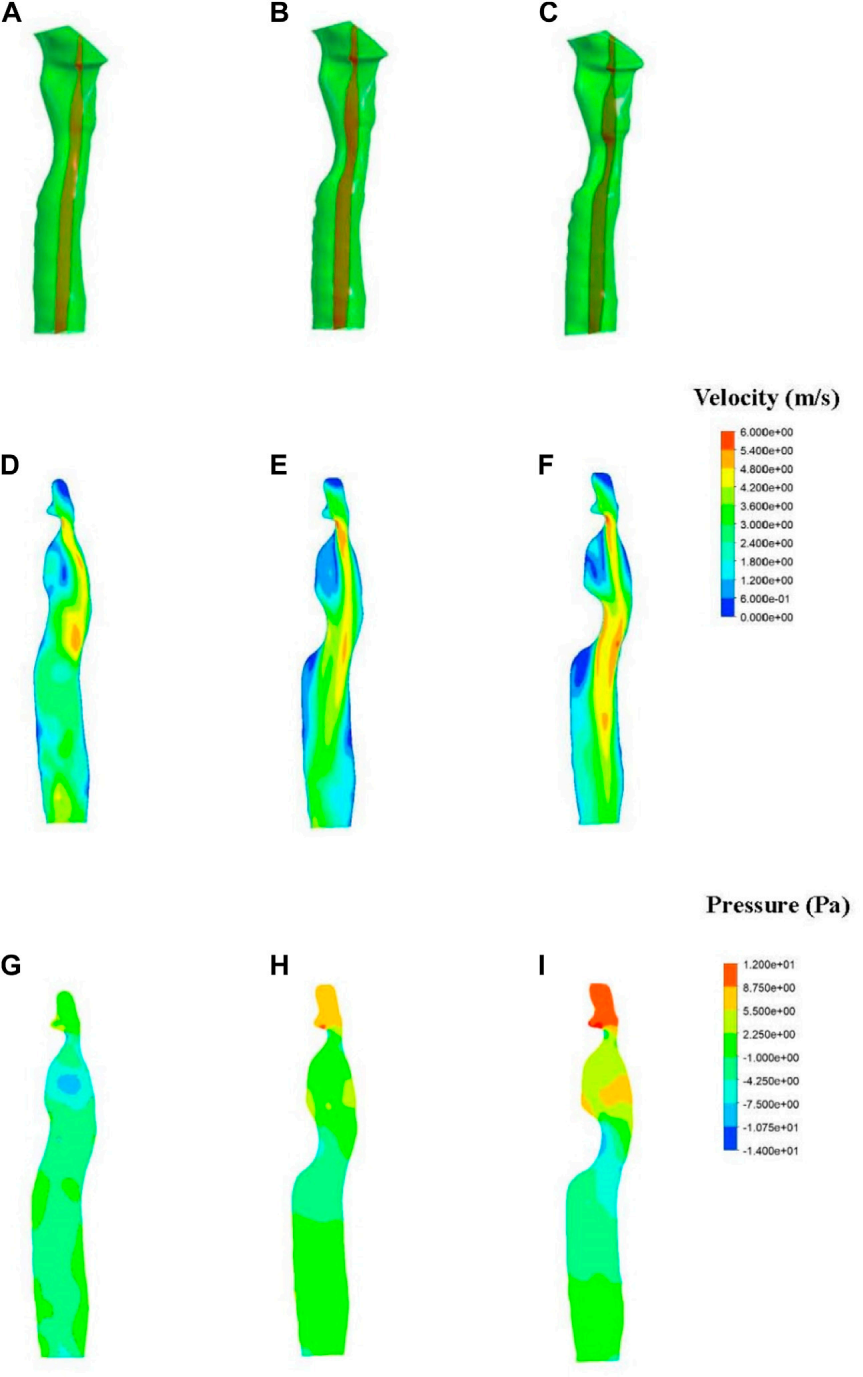
Fluid simulations associated with patient one. **(A–C)** Section position (shown in red). **(D–F)** Contours of velocity distribution. **(G–I)** Contours of pressure distribution.

**FIGURE 6:**
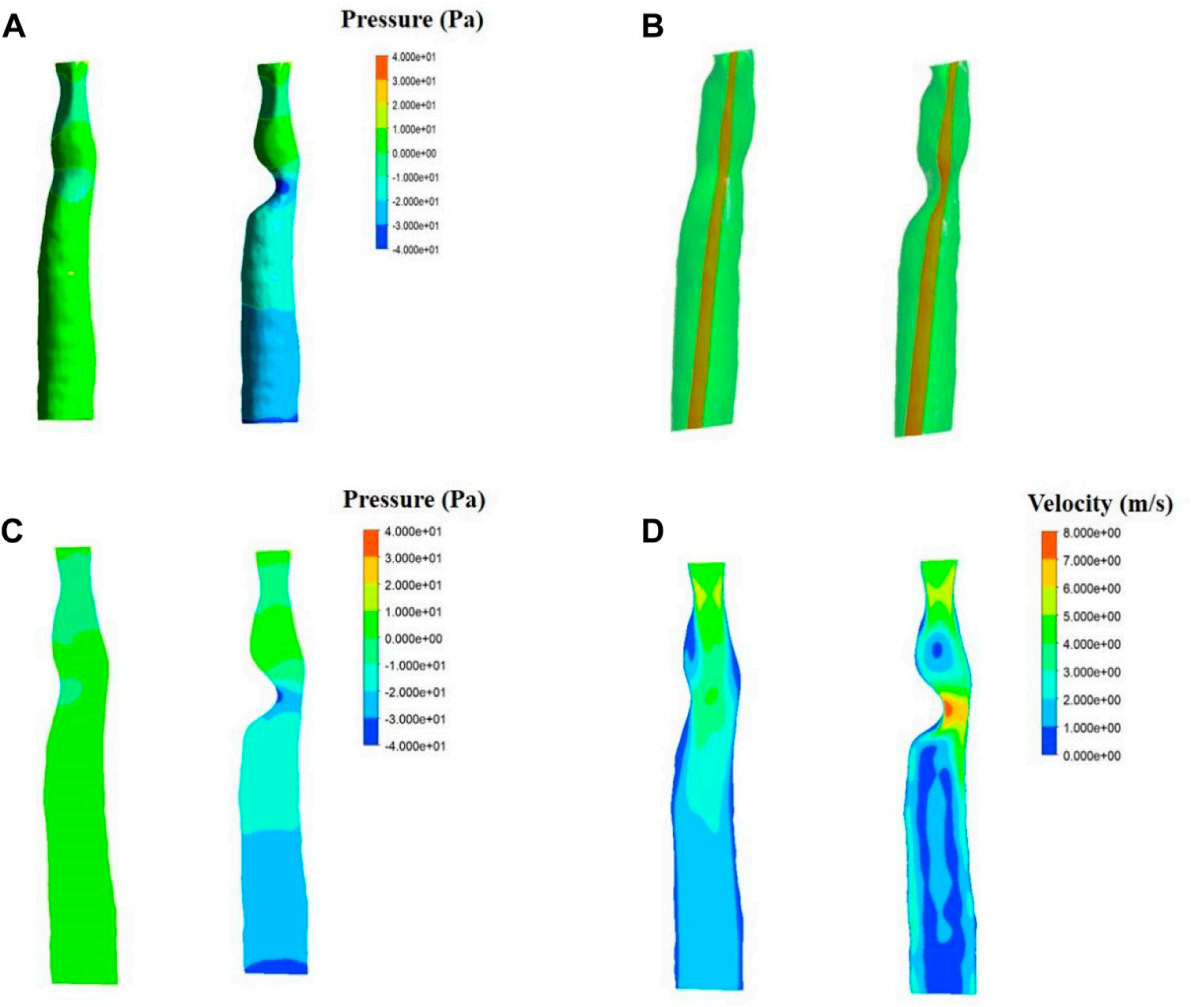
Fluid simulations associated with patient two. Simulations for a normal trachea are on the left side of each panel, while simulations including 50% stenosis are on the right. **(A)**. Results of fluid pressure. **(B)**. Section position (shown in red). **(C)**. Contours of velocity distribution. **(D)**. Contours of pressure distribution.

Results of velocity distribution in the first patient are presented in Figure 5. A higher velocity was observed as the degree of stenosis increased in the local area near the wall of the patch. The velocity of the normal trachea in the first patient was 1.2–3 m/s, while the velocity of the 50% stenosis trachea reached 5.4 m/s in the area near the wall. The velocity distribution of the normal trachea in the second patient was 2–4 m/s, while the velocity of the 50% stenosis trachea reached 8 m/s in the area near the wall (Figure 6D).

### 3.3 Solid simulation results

**FIGURE 7:**
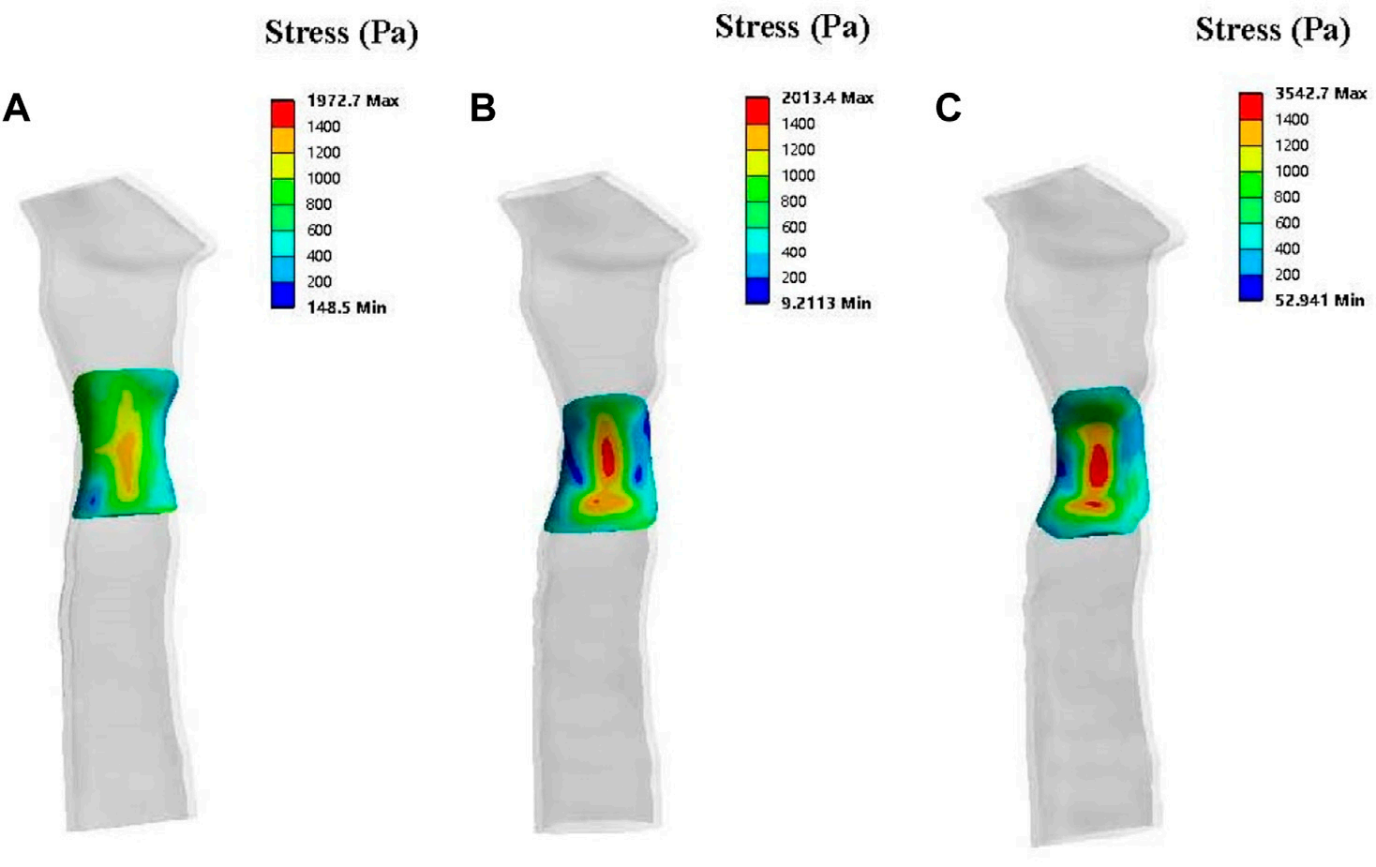
Solid simulations depicting the contours of stress distribution on the patch for patient one. The lateral view of a normal trachea **(A)**, a trachea with 33% stenosis **(B)** and a trachea with 50% stenosis **(C)**.

The stress simulation results from patient one are presented in Figure 7. The stress on the mesh was affected by different degrees of stenosis. A greater level of stress was observed in the central region of the periosteum in the trachea, with 50% stenosis compared to no stenosis and 33% stenosis morphologies. The stress on the normal tracheal wall in the yellow area was 1,000–1,400 Pa, and this area expanded as the degree of stenosis increased. In addition, the maximum stress also increased with the amplification of stenosis.

**FIGURE 8:**
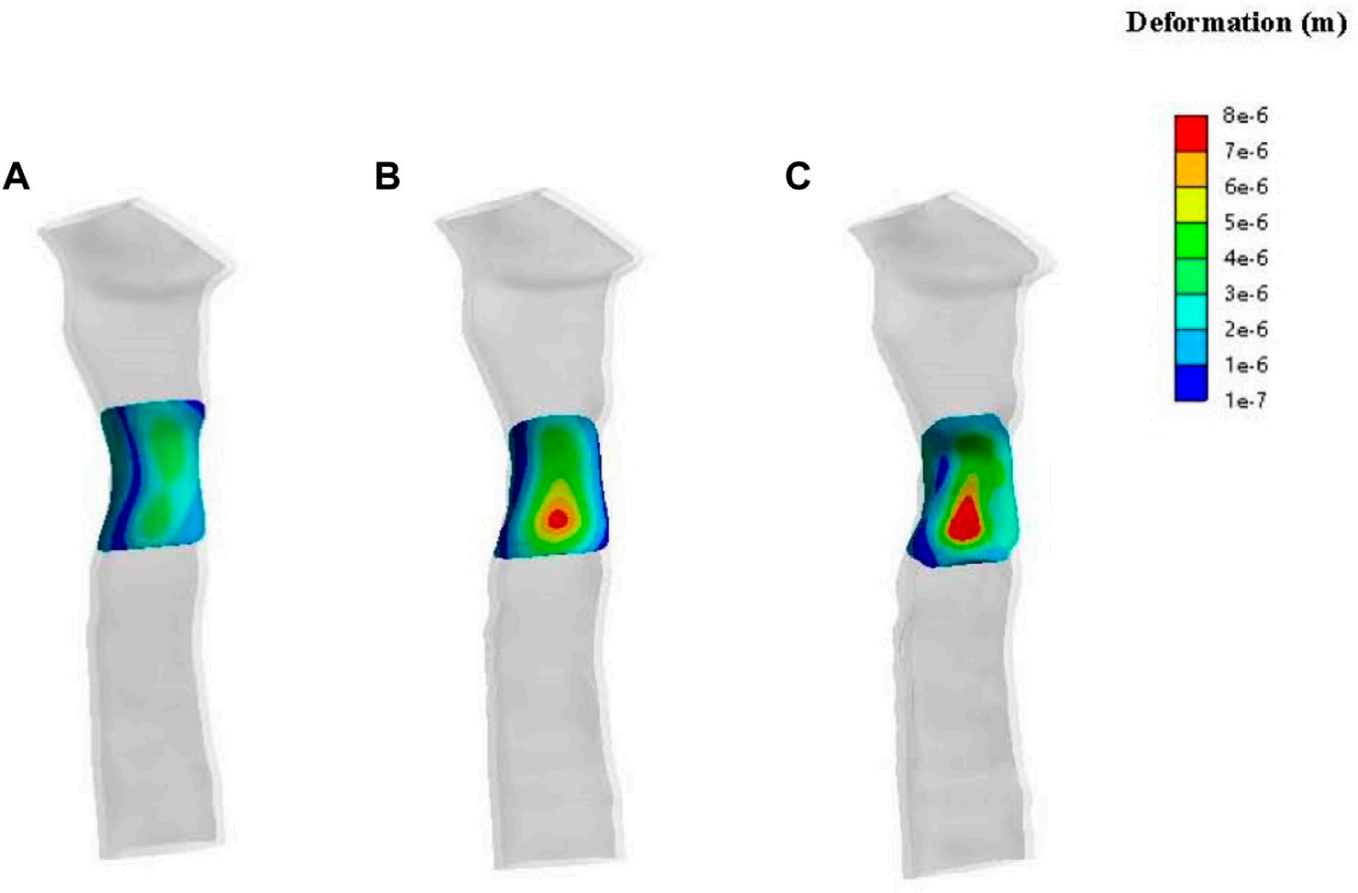
Contours of deformation on the patch for patient one. The lateral view of a normal trachea **(A)**, a trachea with 33% stenosis **(B)** and a trachea with 50% stenosis **(C)**.

Results of patch deformation analysis in patient one show deformations of the various periosteum morphologies at 2 mmHg of pressure (Figure 8). In terms of the number of deformations, none of the three morphologies presented significant gross deformations. Compared to a standard tracheal shape, the deformation distribution characteristics of the central area of the periosteal patch showed concentrated and higher deformation, and the deformation size increased with the degree of stenosis.

**FIGURE 9:**
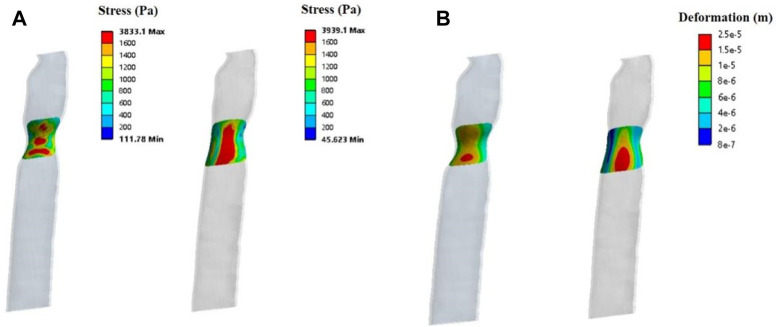
Solid simulations of the patch used for patient two. **(A)**. Contours of stress distribution on the patch. **(B)**. Contours of deformation on the patch.

Patient two’s stress simulation results are shown in Figure 9A. From the perspective of stress size, the stress on the mesh of patient two was more significant than in both the normal and 50% stenosis tracheas of patient one. The stress in the central region (red region) of the periosteum in the normal trachea was 1,600–3,900 Pa and reached a maximum of 3,939 Pa. The maximum stress of the trachea with 50% stenosis in the central periosteum was close to that of the normal trachea, and large pressure areas (red region) are distributed over a more extensive range compared to the normal trachea.

The deformation results of patient two’s patch showed no apparent gross deformation in any morphology. According to the deformation distribution characteristics, the tracheas of the two states in patient two were concentrated, with large areas of deformation in the central region (Figure 9B) and a maximum deformation of 2.5e-5 m. This was more obvious than patient one’s maximum deformation of 8e-6 m.

## 4 Discussion

The sternocleidomastoid myoperiosteal flap, which uses the SCM as the pedicle and the clavicular periosteum as the flap, has been regarded as a suitable material for the reconstruction of non-circular tracheal defects since it was successfully used for tracheal reconstruction in 1983 (Tovi and Gittot 1983). However, since the clavicular periosteum is softer than tracheal cartilage, there is a risk of tracheal stenosis due to a clavicular periosteum collapse (Xia et al., 2021). Therefore, an objective scientific assessment was required to estimate the risk of tracheal stenosis after reconstruction. Biomechanical simulation analysis, which is non-invasive and approaches a physiological state, might be a potential research and evaluation method. This study established a biomechanical tracheal reconstruction model for the first time, and different tracheal widths after reconstruction were evaluated.

A solid mechanical analysis of the trachea revealed that the stress and deformation on the tracheal wall increased with the degree of stenosis. The trachea with 50% stenosis had the most significant wall deformation among the three simulation conditions. Under the same condition of 50% stenosis, the stress and deformation in the trachea wall are greater in patients with smaller normal trachea lumen. The increased stress in the patch may lead to tissue remodeling and wall thickening with consequent granulation and scarring of the repaired trachea.

The fluid simulation showed that the fluid pressure on the periosteal patch area decreased with an increase in tracheal stenosis, while the fluid velocity increased with the stenosis. Although no significant gross deformation on the periosteal region was observed in the solid simulation, lower fluid pressure on the reconstructed tracheal wall, higher velocity of turbulence and higher stress in the patch may cause further deformation of the periosteum in the healing process.

Hence, for patients with smaller tracheal diameters, the risk of tracheal stenosis after reconstruction is much higher than for patients with a wider trachea according to the results of mechanical simulation analysis. The clinical follow-up showed that patients with a greater tracheal diameter immediately after surgery didn’t have stenosis eventually, while patients with a smaller diameter had apparent stenosis 2 months postoperatively. The clinical results proved that besides the periosteal deformation, the fluid pressure, fluid velocity, and solid stress in the repaired trachea might have a noticeable effect during the healing of the reconstructed trachea.

The results of the solid mechanical simulation of the trachea showed no significant gross deformation on the periosteum region during inspiration. This implies that the collapse of the trachea may not happen after a tracheal defect reconstruction larger than 50% of the circumference. Since the reconstructed trachea’s initial cross-sectional area has a significant influence on the local fluid and solid mechanics, the results of this study suggest that it is mechanically advantageous to preserve as much luminal cross-sectional area as possible during surgery.

This study established the first biomechanical model of tracheal reconstruction using clavicular periosteal tensile testing data. The follow-up data from different patients verified the mechanical simulation findings. These strengths provide reliability for the analysis in this study. However, there are some limitations to our research as well. First, this study used linear elastic tracheal cartilage as the material for the tracheal model, which differs from reality. Future studies could consider using a combination of materials to define the material parameters of the tracheal model, especially the muscle tissue at the posterior part of the tracheal wall. Additionally, the harvested periosteum was treated as an isotropic material in the simulation. As the clavicular periosteum is anisotropic in reality, further research on the anisotropic mechanical properties of the clavicular periosteum is required. Furthermore, the periosteum in this study was taken from a male patient, which might be different from the periosteum of female patients. In clinical practice, tracheal stenosis after reconstruction often occurs in female patients, possibly because the clavicular periosteum of females might be softer than that of males. The mechanical properties of the female clavicle periosteum need to be investigated in the future. Finally, the mechanical models of the trachea and clavicular flap constructed in this study ignored the potential influence of prestress. In clinical surgery, surgeons usually harvest a clavicular flap larger than the tracheal defect to avoid prestress. However, due to the complexity of the surgical process and the individual characteristics of the operating area, the clavicular flap may be overstrained in the reconstruction process in rare cases, and the prestress generated in such condition might affect the distribution of stress. The influence of prestress will be taken into account in our further study, and the mechanical state of the clavicular flap will be analyzed individually based on this.

## 5 Conclusion

This study investigated the airflow dynamics and solid mechanics of trachea reconstruction with the clavicular periosteum. Our results showed that 1) the numerical simulation method would provide references for biomechanical evaluation of trachea reconstruction surgery, 2) the flow rate increased and the air pressure on the patch decreased with the decrease of the trachea cross-sectional area, 3) the stress and deformation of the patch increased as the trachea stenosis increased, 4) with the same degree of tracheal stenosis, the larger the initial tracheal cross-sectional area of the patient, the smaller the stress and deformation of the tube wall. To the best of our knowledge, this is the first study to establish a biomechanical model of tracheal reconstruction using the clavicular periosteum, analyzing the fluid and solid mechanical changes in varied degrees of stenosis in patients with different initial tracheal cross-sectional area. Our model provides the possibility of objective evaluation for tracheal reconstruction *via* SCM clavicular periosteal flap. It may expand the surgical indications based on the model prediction results in the future.

## Data Availability

The raw data supporting the conclusion of this article will be made available by the authors, without undue reservation.
